# Sustainable cultivation of phytopharmaceuticals in Baden-Wuerttemberg, Germany: a SWOT analysis and future directions

**DOI:** 10.1080/13880209.2025.2457328

**Published:** 2025-02-01

**Authors:** Peter W. Heger, Ilka Meinert, Peter Nick, Peter Riedl, Michael Heinrich, Michael Straub

**Affiliations:** a Health Research Services GmbH, Germany; b Karlsruher Institut für Technologie, Joseph Gottlieb Koelreuter Institut für Pflanzenwissenschaften, Karlsruhe, Germany; c Salus Haus Dr. med. Otto Greither Nachf. GmbH & Co. KG, Bruckmuehl, Germany; d Pharmacognosy and Phytotherapy, University College London - School of Pharmacy, London, United Kingdom; e Department of Chinese Pharmaceutical Sciences and Chinese Medicine Resources, College of Chinese Medicine, China Medical University, Taichung, Taiwan; f STRAUB INT. ECO-CONSULTING, Mutlangen, Germany

**Keywords:** SWOT analysis, sustainable cultivation, medicinal plant cultivation, *Arnica montana L*. (true arnica;), *hydrastis canadensis L.* (goldenseal/canadian turmeric;), *rheum rhaponticum L.* (Rhapontic rhubarb;), traditional medicine

## Abstract

**Objectives:**

The aim of this systematic work is a Strengths, Weaknesses, Opportunities, Threats (SWOT) analysis to select suitable medicinal plants for cultivation in the region of Baden-Wuerttemberg, Germany.

**Methods:**

A systematic SWOT analysis, based on expert assesments and literature research, was performed considering factors like market demand, cultivation conditions, and potential economic benefits.

**Results:**

Medicinal plants have been essential for producing compounds with significant health benefits. However, unsuitable harvesting practices threaten plant species and traditional communities due to loss of knowledge and culture. In response, sustainable cultivation is gaining attention as alternative to wild collection, ensuring both biodiversity conversation and integrity of medicinal products. Three plants – *Arnica montana L.*, *Hydrastis canadensis L.,* and *Rheum rhaponticum L.* – were identified as particularly suitable due to their high demand and feasibility of their cultivation under local conditions. Conversely, six other plants were deemed less viable due to various challenges, including market competition and harvesting difficulties.

**Conclusions:**

This publication emphasizes the importance of comprehensive planning and analysis in transitioning from wild collection to sustainable cultivation of medicinal plants, highlighting the potential benefits for regional agriculture, conservation efforts, and the pharmaceutical industry. BIOPRO Baden-Württemberg GmbH promotes this approach by fostering a bioeconomy centred on cultivating high-value medicinal plants in the state of Baden-Wuerttemberg, Germany.

## Introduction

Plants are endowed with a very rich secondary metabolism and, therefore, have been a central source for medicinal products from the beginning of humanity and have retained a tremendous economic impact. In the United States alone, the yearly sales of supplements exceeded 7.152 billion US$ in 2021 (Smith et al. 2022), annual growth rates for the trade with medicinal plants are steadily at around 20% (Booker et al. 2012). However, the bioactivity is caused by active ingredients (e.g., essential oils, flavonoids, glycosides, or bitter substances) that derive from usually complex and highly specialized metabolic pathways. These pathways are often active in specific cells, and they are fully unfolded only in specific species, which means that the yield of these compounds is often very low. As a result, phytomedical products may be costly and the respective plants are often threatened by overcollection. For instance, to produce 1 g of the important antitumor compound paclitaxel, 10 kg of the rare and slow-growing tree *Taxus wallichiana Zucc*. are required, which shifted this species within a few years to the verge of extinction (Farjon and Page 1999; Heinrich et al. 2023). The compound was initially identified in *Taxus brevifolia Nutt*. (Californian Yew Tree) (Kewscience 2024a), also a slow growing species. The supply issues concerning taxol were overcome with its semi-synthesis by the conversion of metabolites present in larger amounts (e.g., 10-deacetylbaccatin III) in the needles of the related English yew (*Taxus baccata* L.). Desoxyharringtonin, so far, the most potent ailment for chronic myeloid leukemia needs to be purified from the endemic tree *Cephalotaxus hainanensis H.L.Li* (Kewscience 2024b). The biotechnological production has allowed an upscaling of the manufacturing *via* a synthesis from the stereochemically similar precursor reducing the need for silvicultural production (Qiao et al. 2023). Meanwhile, this tree has become so endangered that it needs to be guarded, because its bark is traded at eight times the price of gold.

Collection of valuable medicinal plants in the wild is not only problematic, because it is not sustainable but may even lead to the extinction of a species. In addition, to find and collect such plants requires considerable expertise, often embedded into cultural contexts of traditional communities that are endangered as well, for instance Sinti and Roma in Europe, or indigenous communities in Assam. As a result, markets are flooded by surrogates or counterfeits. This can be paradigmatically shown for the insect-parasitic fungus *Cordyceps sinensis* widely valued in Asian traditional medicine but extremely rare (Li et al. 2011). A hype of this fungus as ʻsuperfoodʼ in Western countries has depleted the market and boosted the price to around 40,000 US$ within five years (Lo et al. 2013). Meanwhile, the market volume of traded *Cordyceps* exceeds the annual harvest of the true fungus by a factor of 20 (Ichim et al. 2020).

Cultivation of medicinal plants represents an alternative that is not only sustainable, but also ensures authenticity and, thus, standardized quality of the product. However, despite a long tradition and accordingly extensive experience, this strategy is demanding and requires a thorough understanding of the conditions at the natural location and their influence into the accumulation of the value-giving compounds in their functional context. Only when these preconditions are met, it will be possible to build up alternatives to wild collection.

These considerations motivated the establishment of the network ‘Phytopharmaceuticals and high value natural products’ (ʻPhytopharmaka und wertgebende Pflanzeninhaltsstoffeʼ), coordinated by BIOPRO Baden-Württemberg GmbH in Stuttgart (Germany) as part of the Sustainable Bioeconomy strategy of the State of Baden-Wuerttemberg. This Thematic Initiative is focused on phytopharmaceuticals and other high-value natural products from plants, exploring needs, opportunities, and challenges of the local phytopharmaceutical industry and the agricultural sector along the value chain (Phytopharmaceuticals and valuable plant ingredients - opportunities for industry and agriculture in Baden-Württemberg - Biopro BW). While being a regional initiative, the work areas of this initiative are of general economic relevance because it explores novel opportunities for agriculture and horticulture supported through partnerships with participants with diverse areas of expertise (The Phytopharmaceuticals and Valuable Plant Ingredients initiative - Biopro BW).

Baden-Wuerttemberg is located in the southwestern part of Germany. To its west lie the Rhine River and the Black Forest, while to the south are Lake Constance and the foothills of the Alps. To the east, the Swabian Alb can be found and to the north the Hohenlohe plain and the Kraichgau. Baden-Wuerttemberg’s geology is highly diverse: the Rhine Plain features gravels and sands, while the Black Forest is characterized by gneiss and granite, the high plateau of the Swabian Alb consists of loess and loess loam, and the Kraichgau area contains keuper and shell limestone. Other soil types in the region include but are not limited to red sandstone, carboniferous sediments as well as jura (Regierungspraesidium Freiburg 2024). The climate in Baden Württemberg varies significantly: Warm, sunny days with occasional rain in spring and fall, intense heat in the summer, especially in the Rhine Valley, and snow in winter at higher altitudes (Merkel 2024). Due to the variations in landscape, climate, and soil quality, a wide range of Central European plants can be cultivated.

The aim of this article is to present the utilization of an analysis focusing on strengths, weaknesses, opportunities, and threats (SWOT analysis) in the determination of suitable crops through the example of the cultivation of medicinal plants in the German state of Baden-Wuerttemberg. SWOT analyses have previously been used in the evaluation of suitable plants or cultivation methods in specific regions (Djodjic et al. 2018; Olum et al. 2018; Kaymaz et al. 2022). Earlier publications have described optimistic future expectations with high potential especially for the cultivation of plants to be used for cosmetics, tea, spices, and dietary supplements, where high-quality products from organic farming in Baden-Wuerttemberg were expected to play an important role (Gebhardt 2022). However, they also describe changes in the political framework for homeopathic medicines, leading to uncertainty and confusion among market participants. The SWOT analysis described in this article was performed by an expert panel consisting of members of the Thematic Initiative described above.

## Materials and methods

In order to determine suitable medicinal plants to be cultivated in the state of Baden-Wuerttemberg, our team conducted a SWOT analysis. A SWOT analysis allows for a realistic and reliable assessment of a particular project, business, or decision to be taken (Calicchio 2020). It is used to analyse the status-quo by considering all potential questions and issues that are of relevance for the cultivation and the subsequent value chain. The tool allows for an evaluation of the advantages and disadvantages of a project and is an important cornerstone of the decision-making process. In this paper, the application of a SWOT analysis for a selection of suitable crops for cultivation is presented using nine crops with agronomic potential in the state of Baden-Württemberg. The presented technique can be transferred to other geographical regions.

In the context of agricultural crop selection, a SWOT analysis helps farmers and agricultural planners to assess various factors influencing their crop choices:

1. Strengths: These are internal factors (Pahl and Richter 2009) that are advantageous to the crop selection process. This could include the availability of skilled labour, suitable climate and soil conditions, and access to advanced farming technology.
2. Weaknesses: These are internal factors (Pahl and Richter 2009) that could hinder successful crop selection. It might involve limitations such as lack of expertise, lack of scientific data on optimal production and processing systems, inadequate infrastructure, susceptibility to certain pests or diseases, or poor water management.
3. Opportunities: These are external factors (Pahl and Richter 2009) that could positively impact crop selection. This can arise from emerging market trends, new agricultural technologies, favourable trade agreements, or growing consumer demand for specific crops.
4. Threats: These are external factors (Pahl and Richter 2009) that could pose challenges to crop selection. Threats might include unpredictable weather patterns, market fluctuations, increased competition from other farmers or regions, or regulatory changes affecting crop production or trade.

By analysing these four dimensions, agricultural stakeholders can come to informed decisions about which crops to prioritize (or to exclude), which strategies to adopt, and how to mitigate potential risks. This process aids in developing a comprehensive understanding of the current agricultural landscape and the factors that should guide crop selection for optimal results.

The aim of this article is to present the utilization of a SWOT analysis in the determination of suitable crops through the example of the cultivation of medicinal plants in the German state of Baden-Wuerttemberg. The SWOT analysis, which is described in this article, was performed by an expert panel composed of members of the Thematic Initiative. The sources of data and information used in the SWOT analysis were based on the long-lasting experiences and knowledge of experts of participating academic institutions and companies working in relevant fields (medical plants, agricultural and political environment), all of which are listed in the appendix of this paper. Therefore, experience-based evaluations and discussions as well as quantitative assessments were carried out by an expert panel consisting of representatives along the value chain.

The medicinal plants were assessed during several meetings of the panel. Both, qualitative and quantitative methods were applied. Main scoring criteria were economic needs, market potential, competition, climatic and growing conditions and cultivation requirements. These aspects were rated for all nine crops using a ten-level Likert scale (1 = suitable without restrictions, 10 = completely unsuitable).

## Results and discussion

A list of nine suitable medical plants was selected for potential cultivation in the region of Baden-Wuerttemberg. This initial selection was based on the current demand medicinal plants on the global market, their availability, potential conflicts arising from species protection, and most importantly the regional cultivation opportunities.

As the range of species that can be cultivated in Germany is in the thousands, all soil types, climates and agricultural practices are covered, and the three shortlisted species can all be cultivated within the country (Federal Office of Consumer Protection and Food Safety 2014). Baden Wuerttemberg offers the necessary framework conditions for the cultivation of the selected species (climate, soils, growers with free resources, etc.) (Sucholas et al. 2023). For the cultivation opportunities, potential difficulties related to the time to first harvest, local pesticide regulations, competitive farming, and expected quality of the harvest were considered. The initial situation of these nine different plants was evaluated focusing on how a suitable value chain could be developed. It was a prerequisite for the analysis that each plant can also be cultivated or collected in Baden-Wuerttemberg. Another key component in the evaluation is the economic context, the cultivation options, and the presence of potential buyers/manufacturers. The following medicinal plants used in licensed or registered pharmaceutical products (Heinrich 2015) were evaluated by an expert panel: Arnica, Canadian turmeric, dandelion, field horsetail, hawthorn, nettle, St. John’s wort, Rhapontic rhubarb, and valerian. In a second step, the panel selected the following three out of these nine plants, based on their high relevance for potential cultivation with industrialization potential and need, as identified through a SWOT analysis (see Table 1 for detailed analyses):

**Table 1.** Results of the SWOT analyses of all nine plants which were evaluated by the expert panel.

Strengths	Weaknesses	Opportunities	Threats
Arnica			
Great demand Several crop parts used, different qualities demanded Variety available ARBO, Eickmeyer, Kneipp Known medicinal plant	Special soil conditions needed Suitable habitat necessary Low pH value of soil required High iron levels in soil required Can only be applied externally	Cultivation experiences exist Few growers Strong customer loyalty	Arnica fly Climate change (Applequist et al. 2020) Relatively high allergenic potential
Canadian turmeric			
Cultivation possible Attractive prices, exclusive culture (strong customer loyalty)	First harvest after three years at the earliest, inventory build up takes a long time (>5 years) Root harvest, right germination temperatures needed (long stratification time) Limited market size Varieties needed Cultivation already performed in the US (Davis and McCoy 2020)	Little competition in cultivation, CITES plant Parallels to the ginseng culture (half -shade plant) Still little cultivation Big demand (many processors) yet only small amounts needed from each	Needs shade, moisture, humus soil, delicate culture Vole damage Climate change (Applequist et al. 2020) Limited applications with negligible use in homeopathy Low toxicological potential (Mandal et al. 2020)
Dandelion			
Several crop parts used, leaves and blossoms first, roots later	In the case of foliar crops, timely harvest required before flowering, leading to shorter harvest period Variety situation confusing in large-scale cultivation Mechanical harvesting may be difficult	Industrial use as a substitute for rubber Market for good qualities established Added value through breeding	Water availability important Powdery mildew infestation in unfavorable weather Pesticide loads Price of foreign goods Wild collections Climate change (Applequist et al. 2020) Contamination from neighbouring fields may be problematic
Field horsetail			
Traditional drug Contained in many tea preparations High demand	Accumulates contaminants (heavy metals and pesticides) Varieties needed	Cultivation trials in Hungary Use to strengthen plants Market for organic goods available Cultivation in poor soil possible	Many goods come from wild collection with low prices Unpolluted location extremely important Climate change (Applequist et al. 2020) Contamination of soil by rhizomes for extended periods
Hawthorn			
Several years of use (>25 years) Quality can be assured (ingredients, species)	Perennial occupancy (>10 years) Many years to first harvest Harvesting (mechanization vs. hand picking), as blossom fades within a few weeks Risk of heavy metal accumulation Varieties needed	Many preparations marketed, high needs, great market potential Agroforestry or landscape element, hedges Wind protection, anti-erosion protection Biodiversity, roosts for beneficial insects Different crop parts used (leaves and flowers, fruit)	Loss of quality due to machine harvest High cost Fire blight / caterpillar infestation (certain cultivation elevation required) Limited long-term experience in the cultivation climate change (Applequist et al. 2020)
Nettle			
Stock establishment with plant culture possible Quality can be ensured through cultivation (contaminants, ingredients) Few chop hours required with good stocks (harrow processing) No sowing	High nitrogen requirements Cultivation of young plants only through experienced companies recommended No established varieties in cultivation Harvesting complex, processing infrastructure required Varieties needed	Perennial use Several crop parts used (leaf, herb, roots, fibres, seed) Currently good sales opportunities Dual use as fibre	Locations rather cool, uniform humidity Caterpillar infestation, feeding damage Contaminants (SM) must be considered during monitoring Price competition through wild collection Climate change (Applequist et al. 2020)
Rhapontic rhubarb			
No previous preparatory work by growers Roots, therefore robust and storable Best climatic conditions Independent from the world market High acceptance by local organic farming	Start with smaller volumes Limited application because of specific activity Perennial cultivation (3 – 7 years) Need for research: Growing conditions Ideal harvest time Pest infestation Cultivation techniques Fertilization Identity clarification Higher cultivation costs in Baden-Württemberg	Increasing sales volume, growing demand Fast availability Low investment risk Low cultivation costs Easy care effort Reasonable sales prices Meristem propagation, then vegetative propagation Currently no known current extension in Germany Early control of drug content and quality possible Optimization of active ingredient content	Lack of long-term experience in the cultivation Climate change (Applequist et al. 2020)
St. John’s wort			
Varieties available High yields in good locations (2 cuts possible) Perennial with good cultivation	Extensive cultivation and wild collection abroad	Market need for high quality goods Loyal customers after qualification	Colletotrichum infestation St. John’s wort beetle Risky culture Low prices Climate change (Applequist et al. 2020)
Valerian			
Results from the KAMEL research project available (Forschungsvereinigung der Arzneimittel-Hersteller e.V. 2012) Cultivation instructions exist Type material available Ingredients when using suitable material, reliably accessible	Cleaning the root in heavy soil expensive	Good sales opportunities at low Prices Loyal customers in particular for medicinal product	Strong price competition abroad Ingredient valerenic acid must be controlled Purity, too many foreign elements due to poor cleaning (earth, sand) Climate change (Applequist et al. 2020)

- *Arnica montana L.* (true arnica) Family: Asteraceae (Compositae) (Edwards et al. 2015): The selection of this plant was based on the high demand and a wide range of products/applications, as well as the availability of suitable regions for cultivation. Several crop parts can be processed (above/underground) and cultivation experience already exists.
- *Hydrastis canadensis L.* (goldenseal/Canadian turmeric) Family: Ranunculaceae (formerly in Berberidaceae) (Edwards et al. 2015): Its wide use in phytotherapeutic and homeopathic products forms the basis for its inclusion. An increasing market demand could be detected. Wild collection and cultivation currently take place in deciduous forests or shading systems are used to simulate the light conditions in deciduous forests.
- *Rheum rhaponticum* L. (Rhapontic rhubarb) Family: Polygonaceae (Heger et al. 2023): Cultivation in the state of Baden-Wuerttemberg is possible due to good climatic conditions. An increasing market demand is evident. Cultivation in Baden-Wuerttemberg would enable or simplify the availability of high-quality harvests and allow for authenticity control. Cultivation experience or culture instructions could exist.

The cultivation of the remaining six plants in Baden-Wuerttemberg was found to be feasible but several potential problems were identified through the SWOT analysis (see Table 1 for all analyses). Below is a short summary of the remaining six plants, which were evaluated by the expert panel but not selected as being of high potential cultivation with industrialization potential and need:

- *Crataegus laevigata (Poir.) DC*., *C. monogyna Jacq.* and their hybrids (Crataegus - hawthorn) Family: Rosaceae, (Edwards et al. 2015): Cultivation is made particularly difficult by the long establishment period until harvest and the particular challenges involved in mechanical harvesting. Land use of > 10 years is commonly required for successful harvest and hawthorn has a tendency for enrichment of heavy metals, increasing the requirements for uncontaminated soil and environment, potentially limiting its usability for medicinal purposes.
- *Equisetum arvense L*. (field horsetail) Family: Equisetaceae, (Edwards et al. 2015): The plant has been excluded due to the listed circumstances (SWOT analysis) and its low market price. Field horsetail tends to enrich heavy metals and pesticides, increasing the requirements for uncontaminated soil and environment, potentially limiting its usability for medicinal purposes.
- *Hypericum perforatum L*. (St. John’s wort) Family: Hypericaceae (Clusiaceae) (Edwards et al. 2015): The plant was excluded from further analyses by the expert panel due to its low market price and strongly fluctuating demand, which makes planning difficult. Both, cultivation and wild collection are successfully performed in other countries. Also, the authentication of *Hypericum perforatum* from wild collection is rather complex because many different types exist.
- *Taraxacum sect. Taraxacum* (dandelion) Family: Asteraceae (Compositae) (Edwards et al. 2015): The plant was excluded from further analyses by the expert group, as the demand appears to be concentrated on a few companies only and was therefore not considered for further evaluation. It has been shown that the varietal purity is difficult to maintain in large scale cultivation.
- *Urtica dioica L*. (stinging nettle) Family: Urticaceae (Edwards et al. 2015): This plant was assigned a special status by the expert panel as it is considered an interesting plant which is unfortunately not suitable for industry as no specific need has yet been defined by these parties. Also, authentication and harvest of nettles is considered to be complex, and a dedicated processing infrastructure is needed. If a dual use medicine and fiber is possible it will be more economically.
- *Valeriana officinalis L*. (valerian) Family: Caprifoliaceae (Valerianaceae) (Edwards et al. 2015): The plant was not deemed to be suitable for cultivation in Baden-Wuerttemberg as there is strong market competition with foreign producers who can already produce efficiently and cost-effectively. Also, a change of approval from food to pharmaceutical drug complex has been implemented and the plant is now subject to stricter restrictions. Another hurdle in successfully marketing the plant is that cleaning of the roots can be very elaborate and expensive in heavy soil.

The resulting requirements are transferred to culture systems and field tests can be carried out before cultivation at a larger scale is performed. Practical implications, particularly how the findings can be applied by local farmers and industry stakeholders are implemented as selected plants are test cultivated since 2022 in various regions of Baden-Wuerttemberg by a local certified farmer and a Medicinal plant cultivation farm (Conzelmann 2022). However, there is unfortunately a complete lack of practical research in Baden-Wuerttemberg on medicinal plant species as carried out by the state institutes. Advice is provided by private consultants and professional organizations such as Netzwerk-Kräuter BW e.V. The diverse geology of Baden-Wuerttemberg, coupled with its varied agricultural and political landscape, highlights the importance of initiatives such as BIOPRO Baden-Wuerttemberg to apply the insights gained from the presented SWOT analysis. The SWOT was found to be a suitable tool to assess various factors influencing crop choices and may guide farmers and agricultural planners in the selection of appropriate crops or medicinal plants.

## General discussion

The aim of this paper was to present a SWOT analysis that was applied to select suitable medicinal plants for cultivation in the state of Baden-Wuerttemberg, Germany. While this pilot project was focused on sustainable phytopharmaceutical cultivation in a small region, it delivers valuable information for the global market. The outcomes achieved through the SWOT analysis presented in this paper serve as a model for other regions globally, showcasing potential advantages and disadvantages of the cultivation of different medicinal plants. Thus, the paper presents a strategy, which enables a robust initial decision-making process to develop novel industrial crops and products for use as medicines and related commodities.

With the increasing impact of climate change on agriculture, pilot projects in regions like Baden-Wuerttemberg can provide insights into potential changes to be considered by local industry including changing weather patterns, soil conditions, and crop resilience. Also, understanding the economic aspects of agricultural decisions is crucial. Therefore, even a local point of view can provide valuable information on cost-effectiveness of cultivating the different medicinal plants, providing a clear picture of the return on investment for investors. This economic consideration is essential for global markets and the economic interaction of local markets, as it helps in decision-making to support sustainable agriculture.

### Limitations

While the state of Baden-Wuerttemberg has a relatively large and successful phytopharmaceutical industry, the local market for phytopharmaceuticals can be considered as relatively small and pricing of the harvested goods may be based on different expectations from supplier’s and customer’s perspectives. Therefore, a SWOT analysis like the one presented in this publication may not capture all aspects related to market transparency, which may be of potential relevance for successful selection and cultivation of medicinal plants.

## Conclusions

In conclusion, this paper presents a SWOT analysis applied to select suitable medicinal plants for cultivation in this the region of Baden-Wuerttemberg, Germany. The three plants of *Arnica montana*, *Hydrastis canadensis*, and *Rheum rhaponticum* were identified as particularly suitable. As always, future directions will depend on specific funding to be secured and specific steps are currently being taken, to enable the cultivation of the three species which have been identified using the process. The next actions will include experimental plots to assess the feasibility of production under real life conditions. Since the requirements of the three target species are very different, the implementation is complex in terms of the regions of production and the specific steps for implementation. However, all three species hold strong bioeconomic promise.

Based on the current SWOT analysis, further research is currently conducted as selected plants are test cultivated since 2022 in various regions of Baden-Wuerttemberg by a local certified farmer and a medicinal plant cultivation farm. However, funding of these types of studies is limited and more support from the local government is desired, especially for domestication research.

## Data Availability

No primary data were generated in this project. The authors confirm that the relevant sources for the SWOT analysis are available within this article.

## Appendix: Members of the BioPro initiative

Companies and institutions involved (as of 14th July 2023):

- Baden-Württembergischer Genossenschaftsverband e. V
- Center for Agricultural Technology Augustenberg
- Dr. Willmar Schwabe GmbH & Co. KG
- Health Research Services GmbH
- Karlsruhe Institute of Technology (KIT) - Joseph Gottlieb Kölreuter Institute for Plant Sciences
- Netzwerk Kräuter, Verein zur Förderung des Heil-, Gewürz- und Kosmetikpflanzenanbaus in Baden-Württemberg
- Straub International Eco-Consulting
- University of Heidelberg – Institute of Pharmacy and Molecular Biology
- University of Hohenheim – Centre for Organic Pharming
- University of Hohenheim – Institute of Crop Science | Agronomy
- University College London - UCL School of Pharmacy
- Vertical Farm Tech GmbH
- VivaCell Biotechnology GmbH
- Walter Schoenenberger Pflanzensaftwerk GmbH & Co. KG
